# The Barley Powdery Mildew Effector Candidates CSEP0081 and CSEP0254 Promote Fungal Infection Success

**DOI:** 10.1371/journal.pone.0157586

**Published:** 2016-06-20

**Authors:** Ali Abdurehim Ahmed, Carsten Pedersen, Hans Thordal-Christensen

**Affiliations:** Section for Plant and Soil Science, Department of Plant and Environmental Sciences, University of Copenhagen, Frederiksberg, Denmark; Universita degli Studi di Pisa, ITALY

## Abstract

Effectors play significant roles in the success of pathogens. Recent advances in genome sequencing have revealed arrays of effectors and effector candidates from a wide range of plant pathogens. Yet, the vast majority of them remain uncharacterized. Among the ~500 Candidate Secreted Effector Proteins (CSEPs) predicted from the barley powdery mildew fungal genome, only a few have been studied and shown to have a function in virulence. Here, we provide evidence that CSEP0081 and CSEP0254 contribute to infection by the fungus. This was studied using Host-Induced Gene Silencing (HIGS), where independent silencing of the transcripts for these *CSEPs* significantly reduced the fungal penetration and haustoria formation rate. Both CSEPs are likely required during and after the formation of haustoria, in which their transcripts were found to be differentially expressed, rather than in epiphytic tissue. When expressed in barley leaf epidermal cells, both CSEPs appears to move freely between the cytosol and the nucleus, suggesting that their host targets locate in these cellular compartments. Collectively, our data suggest that, in addition to the previously reported effectors, the barley powdery mildew fungus utilizes these two CSEPs as virulence factors to enhance infection.

## Introduction

Plants and animals are exposed to a wide range of pathogens and parasites in nature. These perpetual interactions have spurred evolution of complex defense mechanisms in the hosts, and the pathogens have evolved diverse means of circumventing these defense mechanisms [1, 2]. Innate immunity consists of at least two main components: Pattern-Triggered Immunity (PTI) (also known as basal defense) and Effector-Trigger Immunity (ETI). The former is activated upon recognition of highly conserved molecular structures in pathogens, known as Pathogen-Associated Molecular Patterns (PAMPs), by surface-localized plant Pattern-Recognition Receptors (PRRs). PTI is induced in all types of plant-pathogen interactions, albeit with different levels of strengths, and it has a broad-spectrum ability to protect against a wide range of pathogens, exemplified by PTI induced by bacterial flagellin and fungal chitin [1, 3]. The second immunity (ETI) is activated by recognition of pathogen effectors by intracellular or plasma membrane receptors [1–4]. In plants, these receptors are encoded by resistance genes (R-genes), and induce race-specific resistance [1, 3, 4]. Adapted pathogens have acquired these effectors to suppress PTI and ETI, and otherwise manipulate the host cell to make it susceptible [1, 3, 4]. Alternatively, pathogens circumvent ETI, for instance by avoiding recognition through mutations that cause amino acid mutations or complete loss of the recognized effectors [3, 4]. Thousands of effector candidates have been predicted based on genome sequence data from many plant pathogens including fungi, bacteria and oomycetes, as well as plant parasitic nematodes and herbivore insects [5–9]. A significant number of the effector candidates has been studied and demonstrated to play a role in virulence. For many of them, host targets and/or cognate R-proteins have been discovered [10].

*Blumeria graminis* f. sp. *hordei* (*Bgh*) is an obligate biotrophic fungus that causes powdery mildew on barley. The *Bgh* genome has been predicted to encode approximately 500 Candidate Secreted Effector Proteins (CSEPs) based on presence of signal peptides for secretion, lack of transmembrane domains and poor homology to known proteins [11, 12]. These CSEPs are often small proteins and most of them group into 73 families [11, 12]. Some of the CSEPs have been experimentally identified in transcriptomic and proteomic studies from barley leaf epidermal strips containing *Bgh* haustoria [13, 14]. The transcriptomics study identified 107 highly expressed CSEP genes, all of which encode a Y/F/WxC motif in the N-terminus of the mature proteins, originally named Effector candidates (Efc) [13]. This motif consists of one of the aromatic amino acids, tyrosine, phenylalanine or tryptophan, a variable amino acid and cysteine [13]. The proteomics analysis discovered 71 small proteins, all expressed in the haustoria, but not in aerial hyphae and conidia [14].

A function in virulence has been verified for some CSEPs using a single epidermal cell-based Host-Induced Gene Silencing (HIGS) assay, where RNA interference (RNAi) hairpin constructs, directed against CSEP genes, are expressed in the attacked host cell [15–19]. For instance, silencing of *CSEP0055* using this technique significantly reduced infection [16]. In addition, CSEP0055 was found to interact with members of the barley defense proteins, PR1 and PR17 [16]. Pliego et al. [17] investigated the function of 50 effector candidate genes using the same technique and revealed that eight of them contribute to infection. Of these, *CSEP0264* (*B**lumaria* *E**ffector* *C**andidate (BEC) 1011*) was found to compromise pathogen-induced programmed cell death [17]. The contribution by another of these eight candidates, *BEC1019*, was confirmed using barley stripe mosaic virus-induced gene silencing [20]. Moreover, BEC1019 was demonstrated to suppress both cultivar specific and non-specific hypersensitive responses induced by AvrPphB from *Pseudomonas syringae* pv. *phaseolicola* and by *Xanthomas oryzae* pv. *oryzicola*, respectively [20]. Recently, we described the contribution of CSEP0105 and CSEP0162 to infection and that both interact with the barley small heat shock proteins, Hsp16.9 and Hsp17.5 [18]. Specifically, CSEP0105 compromised the chaperone activity of Hsp16.9, likely to interfere with plant defense [18]. Additional eight out of 22 studied CSEP genes were revealed to contribute to early infection [19]. The *Bgh CSEP0214* (*BEC2*) has an orthologue in *Golovinomyces orontii*, a causal agent of Arabidopsis powdery mildew. Stable expression of this gene in *Arabidopsis thaliana* caused increased susceptibility to the non-adapted pea powdery mildew fungus, *Erysiphe pisi* [21].

Apart from the above-mentioned *Bgh* effector candidates, the role of many CSEPs remains elusive. In this study, we investigated the function of seven CSEPs using HIGS, and we demonstrate here that CSEP0081 and CSEP0254 contribute to *Bgh* virulence. These two CSEPs are differentially expressed in the haustoria and they are highly up-regulated during and after haustorial formation. Moreover, we found evidence that both CSEPs move freely between the cytosol and the nucleus of barley leaf epidermal cells.

## Results

### CSEP0081 and CSEP0254 contribute to *Bgh* penetration and haustorial formation success

**Fig 1:**
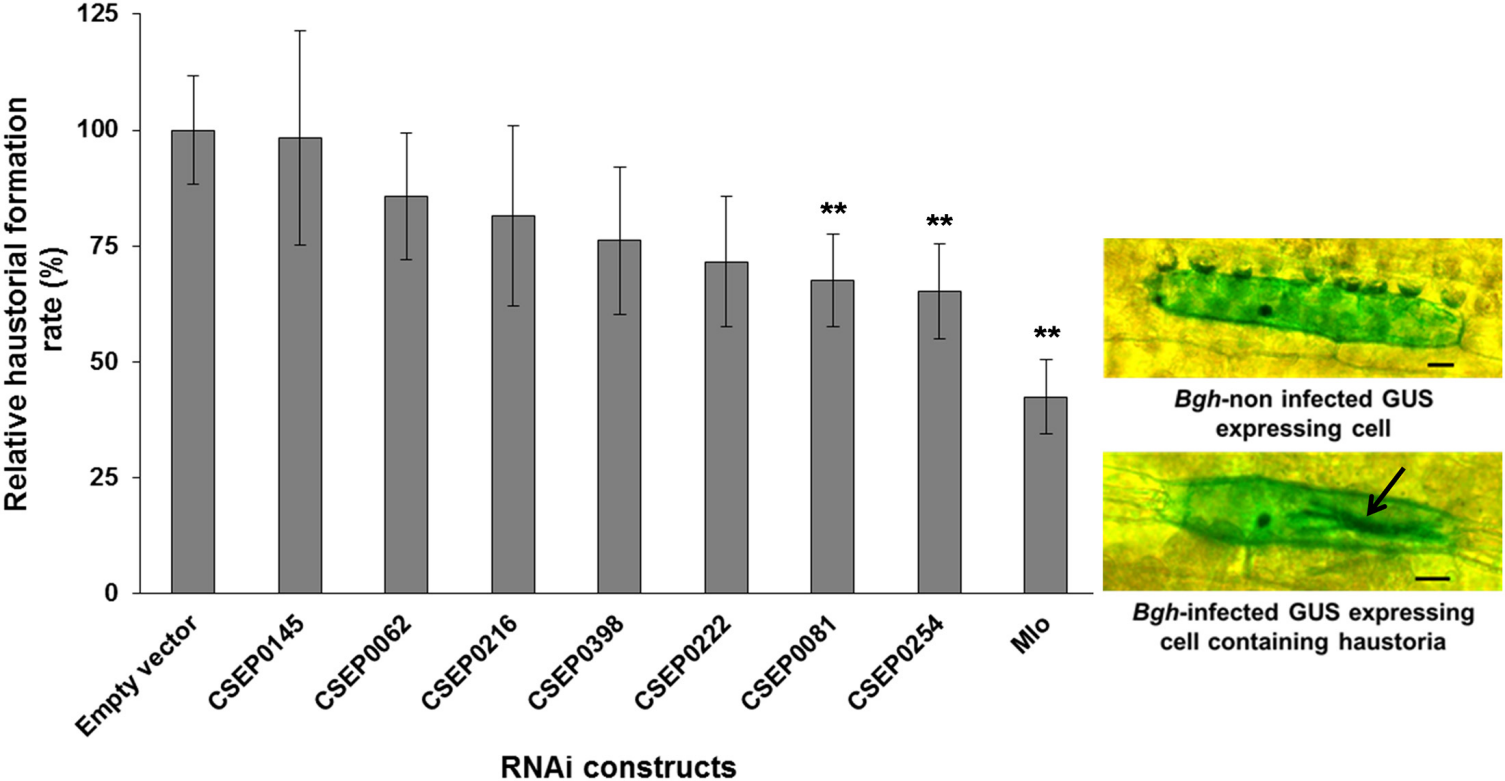
HIGS-mediated silencing of *CSEP0081* and *CSEP0254* decreased *Bgh* haustorial formation success. Barley leaves, bombarded with RNAi and GUS reporter constructs, were infected with *Bgh* and scored for fungal haustoria formation. The haustorial formation rate was calculated as the ratio of haustoria-containing transformed cells (GUS expressing cells) divided by the total number of transformed cells. Relative haustorial formation rate was computed relative to the empty vector control of each experiment, which was set to 100%. Data shown are mean values of five independent experiments, but *CSEP0145*, *CSEP0216*, *CSEP0222*, *CSEP0398* and *Mlo* were excluded from two of those. All values are presented ± SE. ** refers to significant differences compared to the empty vector control (*P* < 0.01). Arrow indicates haustoria. Scale bar, 20 μm.

An essential function of effectors is to promote infection by manipulating host cell defense and metabolism. To determine the role of *Bgh* effector candidates in promoting infection, we took advantage of HIGS [15] and studied a number of CSEPs. From the ~500 predicted CSEPs, seven were chosen (Table 1) representing different CSEP families, expression levels, Y/F/WxC motif sequences as well as the presence or lack of predicted nuclear localization signals [12–14]. The *Bgh* haustorial formation rate was significantly decreased during HIGS-mediated individual silencing of two of the CSEP genes. Approximately 35 and 32% reduction was observed after bombardment with the hairpin constructs for *CSEP0254* and *CSEP0081*, respectively, when compared to the empty vector control (Fig 1 and S1 Table). The *Mlo* RNAi positive control decreased the haustorial formation rate by 58% (Fig 1 and S1 Table).

**Table 1:** Description of the seven selected CSEPs.

Name of CSEP^1^(Efc^2^)	Length (amino acid)^1^	SP length (amino acid)^1^	CSEP family^1^	Contains predicted NLS^1^	Expression level (number of ESTs)^2^	Y/F/WxC motif^2^	Identified from proteomic study^3^
CSEP0062 (8)	160	18	Sim. to 22	-	High (37)	YVC	Haust/hyphae
CSEP0081 (17)	126	27	12	-	High (21)	YNC	-
CSEP0145 (87)	119	24	5	-	Low (2)	YEC	Haustoria
CSEP0216	202	19	48	Yes	-	FNC	-
CSEP0222	408	25	1	Yes	-	FQC	-
CSEP0254 (4)	115	24	8	-	High (52)	YKC	-
CSEP0398	302	20	6	-	-	YLC	Haustoria

^1^CSEP naming according to [12], CSEPs are grouped into 73 families, SP and NLS refer to predicted signal peptide and nuclear localization signal, respectively.

^2^Effector candidate (Efc) naming according to [13], providing numbers of CSEP ESTs out of approximately 10,000 ESTs in the library obtained using Sanger sequencing from haustoria-containing barley leaf epidermal strips 7 days post inoculation. Y/F/WxC motif consists of one of the aromatic amino acids, tyrosine, phenylalanine or tryptophan, a variable amino acid and cysteine.

^3^Comparative study of sporulating hyphae and haustorial proteome [14].

To predict whether the RNAi constructs caused off-target silencing effects on other *Bgh* transcripts, we used the SI-FI software tool (http://labtools.ipk-gatersleben.de/) against the *Bgh* transcriptome. The *CSEP0254* RNAi construct, which caused a significant reduction in the haustorial formation rate, was predicted to additionally target *CSEP0333*. Both CSEP0254 and CSEP0333 belong to family 8. However, RNA-sequencing expression data of *Bgh* isolate DH14 show that the expression level of *CSEP0254* in haustoria is about ten times higher than that of *CSEP0333*. The expression of both *CSEPs* in the epiphytic tissue is low (S1 Fig). In addition, there is no expression data for *CSEP0333* in the RNA-seq analysis of *Bgh* isolates A6 and K1 [22], which must be due to lack of reads. These observations support that the HIGS effect mainly should be attributed to CSEP0254, but does not exclude that CSEP0333 may play a role. However, the highly similar transcript sequences indicate that it would be very difficult to make RNAi constructs targeting these two CSEPs individually (S2 Fig). The sequence of *CSEP0398* RNAi construct suggests that it also targets nine other CSEP genes, namely *CSEP0015*, *CSEP0175*, *CSEP0176*, *CSEP0192*, *CSEP0230*, *CSEP0397*, *CSEP0434*, *CSEP0466* and *CSEP00467*, all of which encode CSEPs of family 6. Surprisingly, no effect on haustorial formation rate was noted for the *CSEP0398* RNAi construct, regardless of nine *CSEP*s potentially being co-silenced. None of the RNAi constructs were predicted to have off-targets in the barley transcriptome (version 12, released on 2011_03_19) as determined by the siRNA scan software (http://bioinfo2.noble.org/RNAiScan.htm). In summary, the reduced haustorial formation rates recorded after individual silencing of the respective *CSEP*s suggest that CSEP0081 and CSEP0254 contribute to infection, most likely as effectors.

### *CSEP0081* and *CSEP0254* are dramatically up-regulated during haustorial formation and secondary penetration

**Fig 2:**
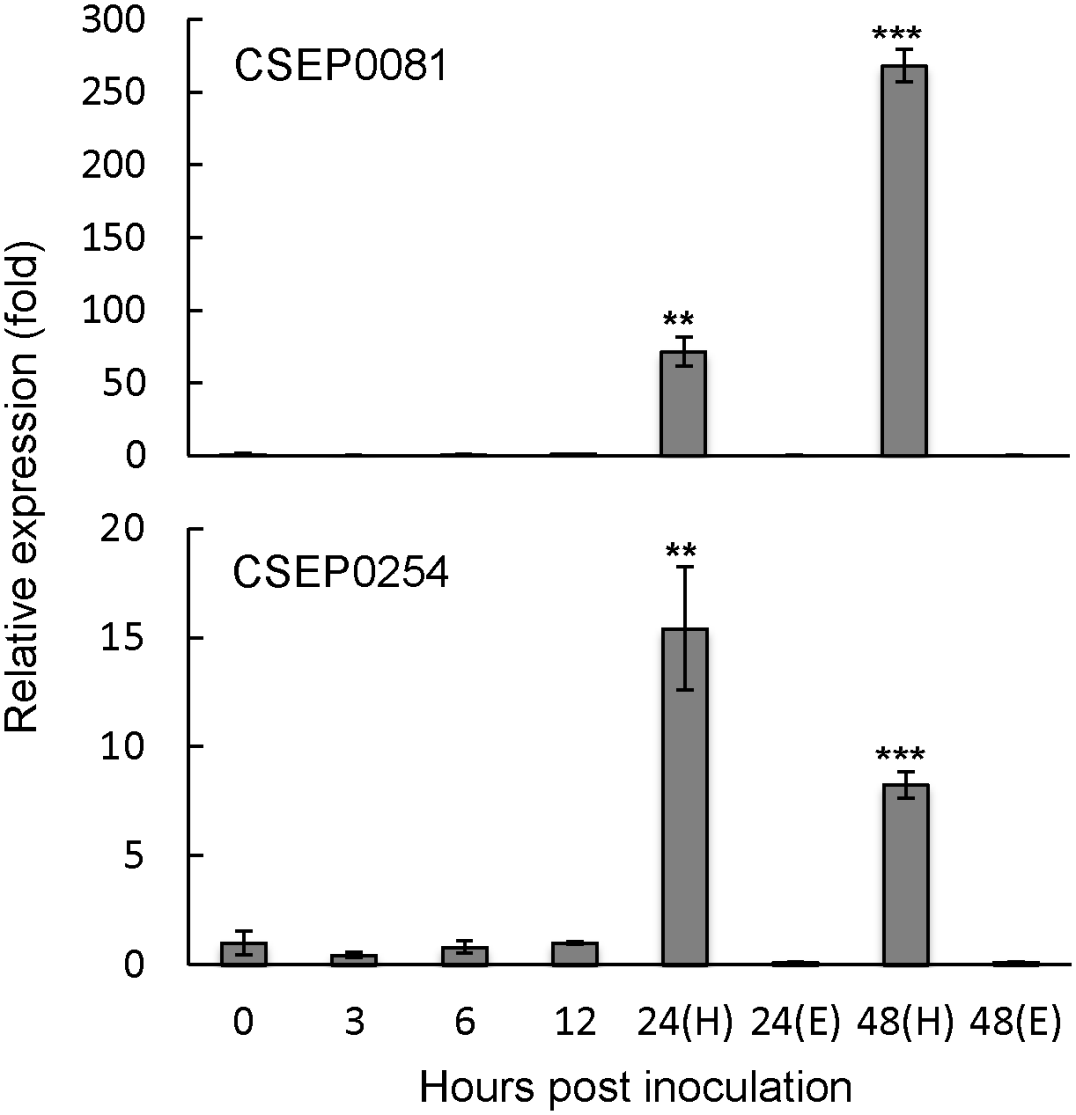
Relative expression levels of *CSEP0081* and *CSEP0254* during *Bgh* infection. The *CSEP* transcripts were monitored using RT-qPCR. Total RNA was isolated from *Bgh*-infected barley leaves. Haustorial and epiphytic expression was analysed separately at 24 and 48 hpi (H and E, respectively). Expression of *Bgh glyceraldehyde 3-phosphate dehydrogenase* (*GAPDH*) was used to normalize the *CSEPs* expression in each sample. Expression was determined relative to 0 hpi, arbitrarily set to one. Data represent means of three independent biological repeats, each with two technical repeats, ± SE. Statistical analysis of biological repeats: *t* test relative to 0 hpi; **, P<0.01; ***, P<0.001.

Pathogens have been shown to employ specific effectors to interfere with the different layers of plant defense triggered during the infection process [1–3]. To suggest the infection stages where *Bgh* effector candidates are likely involved, we studied the expression pattern of *CSEP0081* and *CSEP0254* by quantitative polymerase chain reaction (qPCR) in a compatible *Bgh*/barley interaction at six *Bgh* developmental and infection stages. These stages represent non-germinated conidia (0 h post inoculation [hpi]), primary germ tube formation (3 hpi), appressorial germ tube formation (6 hpi), penetration (12 hpi), haustorial formation (24 hpi) and secondary penetration (48 hpi). For the first four stages, the *CSEP* transcript expression was analyzed by harvesting the entire *Bgh*-infected leaves together with the epiphytic fungal tissues as one entity. However, for the latter two stages, the *CSEP* expression in the epiphytic fungal material and in the haustoria was analyzed separately by harvesting the respective materials one-by-one. The expression profiling indicated that the transcripts of both *CSEPs* were only marginally detectable during the early time points (0–12 hpi) and in the epiphytic material of the last two infection stages, but highly abundant in the leaf samples containing the haustoria (Fig 2 and S2 Table). At 24 hpi, abundance of the *CSEP0081* and *CSEP0254* transcripts were increased by 70 and 15 fold in the haustoria relative to 0 hpi, respectively (Fig 2). At 48 hpi, the transcript levels of *CSEP0081* were further elevated to 270 fold, whereas *CSEP0254* transcript decreased to 8 fold (Fig 2). The haustorial expression levels of these CSEP transcripts were statistically different (P<0.001) from the levels in the epiphytic material at the corresponding time-points. Therefore, it is likely that these CSEPs play a role during and after haustorial formation.

### Subcellular localization of CSEP0081 suggests that the host cytosol and nucleus are the targeted compartments

**Fig 3:**
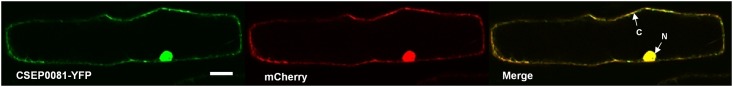
CSEP0081 fused to YFP localizes to the cytosol and the nucleus of barley leaf epidermal cells. Construct encoding CSEP0081, lacking signal peptide, with YFP fused to its C-terminus was co-expressed with a free mCherry marker construct in barley leaf epidermal cells using particle bombardment. Localization was monitored 1 to 3 days later. The CSEP0081-YFP fusion proteins co-localized with mCherry in the cytosol and the nucleus, shown by a complete merge of yellow and red signals. C, cytosol. N, nucleus. Scale bar, 20 μm.

Effector localization in the host cell is mostly correlated with their target proteins and gives relevant information regarding their mode of action [23]. To gain insight where CSEP0081 locates in the host cell and execute its role in virulence, its subcellular distribution was examined in a similar way as in previous studies [23]. Here we used particle bombardment-mediated transient expression of CSEP0081, without its signal peptide and with YFP (yellow fluorescent protein) fused to its C-terminus, in barley leaf epidermal cells, and co-expression with a free mCherry (red fluorescent protein) marker, which localizes to the cytosol and the nucleus. Confocal images appeared to show that the YFP and mCherry fluorescences had complete co-localization in the cytosol and the nucleus (Fig 3). A similar observation has previously been made for a CSEP0254-YFP fusion protein [18]. The co-localization was confirmed by signal quantification showing that the relative intensity of YFP and mCherry in the cytosol and in the nucleus were identical for CSEP0081 as well as for CSEP0254 (S3 Fig). This suggested that both effectors moved freely between the cytosol and nucleus, as did mCherry.

## Discussion

So far, functional studies have evidenced a role in virulence of 19 *Bgh* effector candidates [16–20]. Yet, the contribution of many candidates in promoting infection remains unclear. In this study, we examined the role of seven CSEPs using HIGS and found that two are important for infection. CSEP0081 and CSEP0254 have very low sequence similarity and belong to CSEP families 12 and 8, respectively [12]. Functional studies of *Bgh* genes have been hindered by the absence of a stable *Bgh* transformation protocol. This challenge was eased by the discovery of HIGS. Although the exact mechanism remains to be elucidated [24], different studies have verified the reliability of HIGS to investigate the role of fungal genes in the *Bgh*/barley interaction [15–19] and other pathosystems [25–27]. As an alternative to the single cell-based HIGS assay, virulence function of BEC1019 was demonstrated using BSMV-mediated gene silencing in a whole leaf assay [20]. The roles of in total 21 *Bgh* effector candidates were shown by HIGS ([16–19] and present study).

On the other hand, these HIGS studies failed to reveal a contribution to infection of 61 effector candidates, including the five in this study [17, 19]. If this reflects genuine lack of contribution, the results may suggest that the candidates have minor or no role during the early stages of infection. Alternatively, they could be involved in nutrient uptake and fungal growth, which might not be properly quantified in the single cell-based HIGS assay. It can only evaluate the performance of *Bgh* during the first 2 to 3 dpi, in which time nutrient uptake differences hardly can be revealed. Another possibility could be that some CSEPs may target the same host component. Thus, there might be redundancy of functions. Otherwise, lack of detectable effect could be due to technical limitation of the HIGS method, such as ineffective RNA silencing, or that some effector candidates might function early in the host/pathogen interaction for which the siRNA generated from the HIGS construct is transferred too late to have silencing effect in the fungus.

*Bgh* penetration and haustorial formation rate reductions of 30–70% have been obtained after silencing of individual effector candidates ([16–19] and present study). However, it remains a question how silencing of a single candidate can influence *Bgh* this much, knowing that the pathogen has approximately 500 effector candidates. First of all, we cannot entirely exclude that these effects are due to limitations of the HIGS method, including a possible influence on the host cell, which thereby become resistant, or that the siRNA signal is spreading to other transcripts either in the host or the fungus, thereby amplifying the effect. Having said this, a possible reason could be that *Bgh* effector candidates, activating a host process that support invasion may function in a sequential manner, and that silencing of a leading CSEP among these will abolish the entire chain of events and have a strong impact on the fungus. Another possibility could be that some CSEPs might target and inhibit host proteins, which have a key role in defense. CSEP0055 may be a good example for this hypothesis. Silencing of it reduced fungal penetration rate by 40% and it was shown to interact with the barley PR1 and PR17, which are extracellular candidate proteases [16]. Prior secretion of CSEP0055 will likely inhibit these proteases and create a conducive environment for the other CSEPs, which otherwise might be degraded. Another example is CSEP0105, which compromised the chaperone activity of the barley Hsp16.9. Silencing of this CSEP also reduced the haustorial formation rate by 40% [18]. Small heat shock proteins like Hsp16.9, have potential to stabilize several intracellular proteins, including proteins important for defense, why effector-inhibition of Hsps can be envisioned to have dramatic consequences.

During the compatible interaction between *Bgh* isolate DH14 and the barley cultivar, Golden Promise, the *CSEP0081* and *CSEP0254* transcripts showed almost similar expression patterns. Both had very poor expression in the epiphytic fungal structures, but were highly expressed in the haustoria. This suggests that CSEP0081 and CSEP0254 positively affect haustorial performance and secondary penetration. These expression profiles were in agreement with data from the *Bgh* isolates A6 and K1. This data were obtained from RNA-sequencing analysis during compatible and incompatible interactions between *Bgh* and the partially immuno-compromised *A*. *thaliana* triple mutants, *pen2 pad4 sag101*, with and without the MLA1 immune receptor [22]. However, *CSEP0254* was expressed at a significantly higher level in isolate A6 than in K1 [22]. In the present HIGS experiments, haustorial formation rates were monitored at 72 hpi, and it was not possible to distinguish between primary and secondary penetrations. However, the HIGS data and the expression data together could suggest that CSEP0081 and CSEP0254 play roles related to haustorial performance affecting secondary penetrations, and not the primary penetrations.

We over-expressed fluorescent fusions of CSEP0081 and CSEP0254 and our data indicate that they localize to the barley cell cytosol and nucleus, suggesting that these CSEPs have potential targets in these compartments. Several effectors from different types of pathogens, including fungi, oomycetes, bacteria and virus as well as nematodes, are delivered to the plant cell cytosol and nucleus [10, 28]. For example, 70% of 48 tested *Hyaloperonospora arabidopsidis* effector candidates localize to the plant cytosol and/or the nucleus, suggesting that the targets of these effectors are located here as well [23]. This agrees with the fact that the plant cell cytosol and the nucleus are the centers of several immuno-related activities, performed by resistance and defense-related proteins, including transcription factors and regulators [29, 30].

In conclusion, we provide evidence that CSEP0081 and CSEP0254 act as effectors by promoting *Bgh* infection success. These CSEPs could be required during and after haustorial formation and they could target defense-related proteins that locate in the host cytosol and/or in the nucleus. Previously, potential barley targets were identified for CSEP0055, CSEP0105, CSEP0162 and two other *Bgh* Effector Candidates (BEC), BEC3 and BEC4 [16, 18, 21]. BEC3 was found to interact with a thiopurine methyltransferase [21]. Meanwhile, BEC4 interact with a ubiquitin-conjugating enzyme and an ADP ribosylation factor-GTPase-activating protein (ARF-GAP); likely to hamper proteasomal ubiquitin-mediated degradation pathway and defense-related vesicle trafficking, respectively [21]. Yet, little is known about the identities and functions of host targets for the grand majority of the *Bgh* effector candidates, including CSEP0081 and CSEP0254. Thus, further investigations are needed to address this issue, which will advance our understanding of how *Bgh* control the host cellular machinery at the molecular level to favor its virulence.

## Materials and Methods

### Plant and Fungus

One-week-old seedlings of barley (*Hordeum vulgare*, cultivar Golden Promise) and the virulent isolate, DH14, of the barley powdery mildew fungus (*Blumeria graminis* f. sp. *hordei*) were used for HIGS, expression analysis and localization studies. Barley seedlings were grown at 16 h light (20°C, 150 μEs^-1^m^-2^)/8 h darkness (15°C). The fungus was propagated on cv. Golden Promise by weekly inoculum transfer, simply by dusting the conidia on one-week-old seedlings.

### Cloning Procedures

For the RNAi constructs, 300–375 bp coding sequences of the following effector candidates were PCR-amplified on fungal cDNA, generated from *Bgh-*infected barley leaves, using the primer pairs described in S3 Table. The effector candidates are CSEP0062 (BGHDH14_bgh02836), CSEP0145 (BGHDH14_bgh03736), CSEP0216 (BGHDH14_bgh04781), CSEP0222 (BGHDH14_bgh04929), and CSEP0398 (BGHDH14_bgh04078), for which sequences are available in the EnsemblFungi database (http://fungi.ensembl.org/index.html). The PCR products were TOPO-cloned into the pCR8/GW/TOPO vector (Invitrogen). Such entry clones had already been generated for CSEP0081 (BGHDH14_bgh03006) and CSEP0254 (BGHDH14_bgh05751) [18]. Using Gateway LR clonase reactions (Invitrogen), the seven inserts were transferred to the 35S promoter-driven hairpin destination vector, pIPKTA30N [31].

For the localization constructs, the coding sequences of CSEPs lacking signal peptide and stop codon were PCR amplified on fungal cDNA using the primer pairs listed in S3 Table. The PCR products were TOPO-cloned into the pENTR/D-TOPO vector. Subsequently, the inserts were transferred to a ubiquitin promoter-driven over-expression vector to fuse YFP to the C-terminus of the CSEPs using Gateway LR clonase reactions. All entry and destination constructs were confirmed by sequencing.

### HIGS

Gene silencing in *Bgh* was performed using the host-induced gene silencing method described by Nowara et al. [15]. The RNAi construct and a β-glucuronidase (GUS) reporter gene construct were co-transformed into barley leaf epidermal cells using particle bombardment. Two days later, the leaves were inoculated with *Bgh* (≈ 200 conidia/mm^2^) and after an additional 3 days, they were stained for GUS activity. Fungal haustoria formation was examined in the transformed (GUS expressing) cells using light microscopy. Haustorial formation rate was calculated as the ratio of transformed cells with haustoria divided by the total number of transformed cells. The empty vector pIPKTA30N and the Mlo-RNAi (pIPKTA36) constructs were used as negative and positive controls, respectively. Relative haustorial formation was calculated relative to the empty vector control in each experiment (set to 100%). The data were analysed using the software package SAS (version 9.4, SAS Institute, Cary, NC), by logistic regression (PROC GENMOD, corrected for over-dispersion), using a generalized linear model and assuming a binomial distribution. The probability of *Bgh* haustoria formation was modelled as a linear function of an intercept parameter, a CSEP effect and an experiment effect. Significant differences between each construct and the empty vector RNAi were analysed using Pearson's Chi-Square test.

### Localization

The localization constructs (7 μg) and a mCherry marker construct (7 μg) were transiently co-expressed into barley leaf epidermal cells using particle bombardment according to Douchkov et al. [31]. One to 3 days later, fluorescent images were recorded using a Leica SP5 confocal laser scanning microscope. Lasers of 514 and 543 nm were used to excite YFP and mCherry, respectively. Emissions were detected at 524–539 nm and at 590–640 nm, respectively. The mCherry marker was used as a control that localizes to the cytosol and nucleus.

### RNA isolation, RT-qPCR and quantitative PCR

Total RNA was extracted from *Bgh*-infected (isolate DH14) barley leaves (cultivar Golden Promise) at 0, 3, 6, 12, 24 and 48 hours post inoculation (hpi) using the polyvinylpolypyrrolidone method [32]. The entire infected leaves were used to isolate total RNA for the first four time points, whereas RNA from the epiphytic fungal tissue and the leaf, including haustoria, was extracted separately at 24 and 48 hpi. This was achieved by dipping *Bgh*-infected leaves in 10% (w/v) cellulose acetate in acetone. The leaves were dried for 10 min and the cellulose acetate strips, containing the epiphytic fungal tissues, were separated from the leaves. The SMART MMLV RT kit (Clonthech) was used to synthesize cDNA according to the manufacturers’ instruction using *CSEP*-specific primers (S3 Table). Transcript quantification was performed on a Stratagene MX3000P real-time PCR detection system using the FIREPol^®^ EvaGreen^®^ qPCR-kit (SOLIS BIODYNE). Twenty microliter reactions composed of 4 μL of 5x HOT FIREPol EvaGreen qPCR Mix Plus, 2 μL of cDNA, 1 μL of 10 mM of each gene-specific primer and 12 μL of water were set up. Reactions for each *CSEP* and the reference gene glyceraldehyde 3-phosphate dehydrogenase (GAPDH) (NCBI: X99732.1) were combined in one 96-well plate. Quantitative PCR was carried out using the following thermo cycle: 15 min at 95°C, followed by 40 cycles of 15 s at 95°C, 20 s at 58°C, and 20 s at 72°C. The dissociation curves of the PCR products were recorded between 55°C and 95°C. Amplification efficiencies of the reference gene and genes of interest were between 90% and 100%. The Stratagene MX3000 qPCR software was used to analyze the result. Three biological, each based on two technical repetitions, were included for each time-point. Statistical analysis of the biological repeats was made using Student’s *t* test relative to the 0 h time-point.

## Supporting Information

S1 Fig Epiphytic and haustorial mean expression of *CSEP0333* and *CSEP0254*. Expression data was obtained from Pedersen et al. [12], who determined them by RNA-sequencing of epiphytic material and haustorial epidermal strips at 5 dpi.
(TIF)

S2 Fig Nucleotide sequence alignment of *CSEP0333* and *CSEP0254*. Nucleotides 1–212 belong to the 5’ untranslated region. The remaining are coding sequences. Dots and shades indicate identical and different nucleotides, respectively. Underlined nucleotides are start and stop codons.
(TIF)

S3 Fig Signal intensity of CSEP0081-YFP, CSEP0254-YFP and mCherry marker in the nucleus and cytosol. Cross-sectional line was drawn over the nucleus (1) and cytosol (2), and signal was quantified using the Leica microsystems LAS AF image analyzing program. The CSEP-YFP and mCherry signals show overlapping intensity in the nucleus and cytosol. Scale bar, 20 μm.
(TIF)

S1 Table Haustoria count and statistical analysis of HIGS. (DOCX)

S2 Table Normalized RT-qPCR CSEP transcript quantification. (DOCX)

S3 Table List of forward (F) and reverse (R) primers for plasmid construction and gene expression assay (5´ to 3´). (DOCX)
