# Supplementary figures and images for: The Barley Powdery Mildew Effector Candidates CSEP0081 and CSEP0254 Promote Fungal Infection Success
Source: PLoS One. 2016 Jun 20;11(6):e0157586. doi: 10.1371/journal.pone.0157586 (PMC4913928; doi:10.1371/journal.pone.0157586)

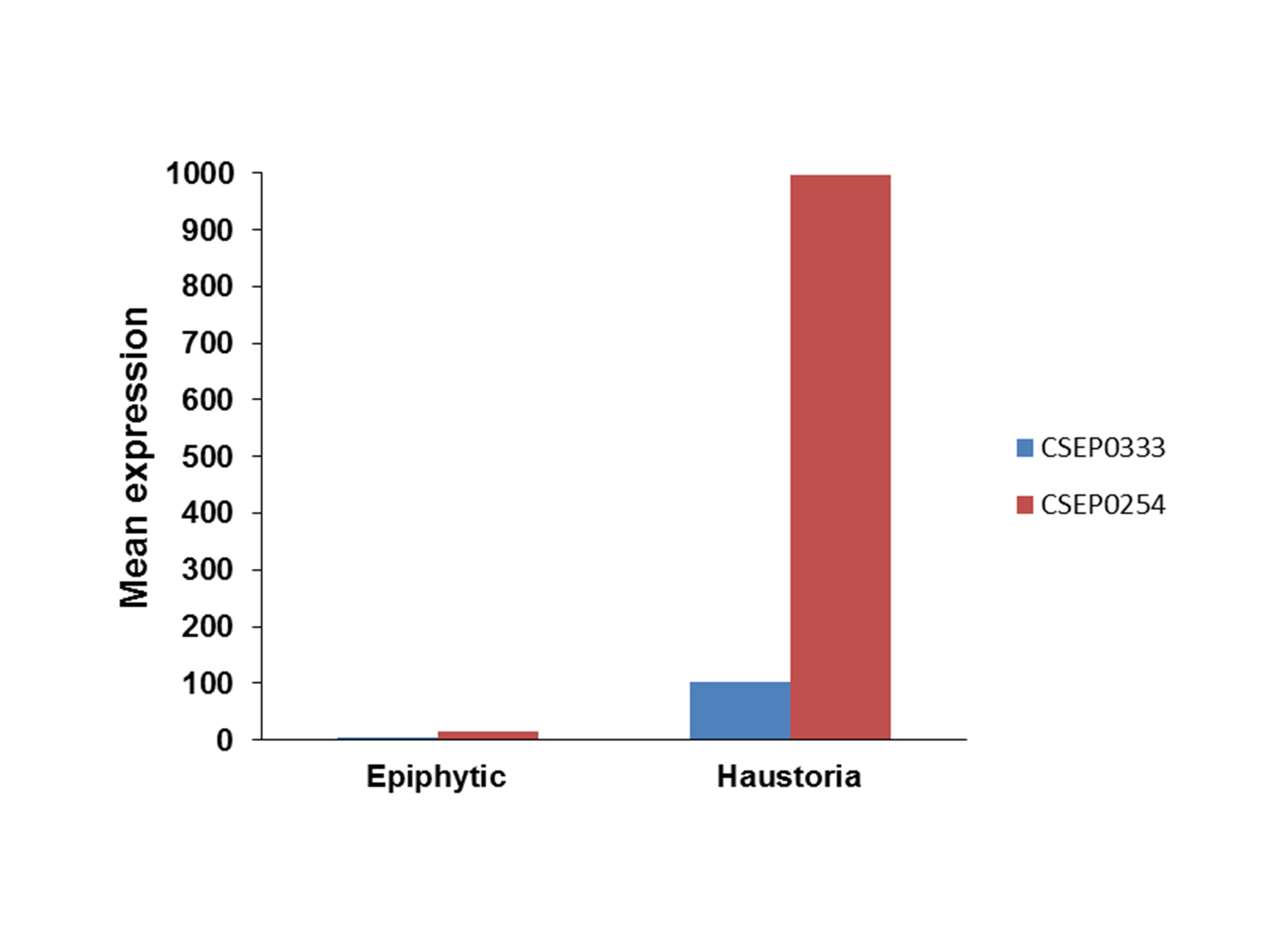

Supplement: S1 Fig — Expression data was obtained from Pedersen et al. [12], who determined them by RNA-sequencing of epiphytic material and haustorial epidermal strips at 5 dpi. (TIF) [file pone.0157586.s001.tif]

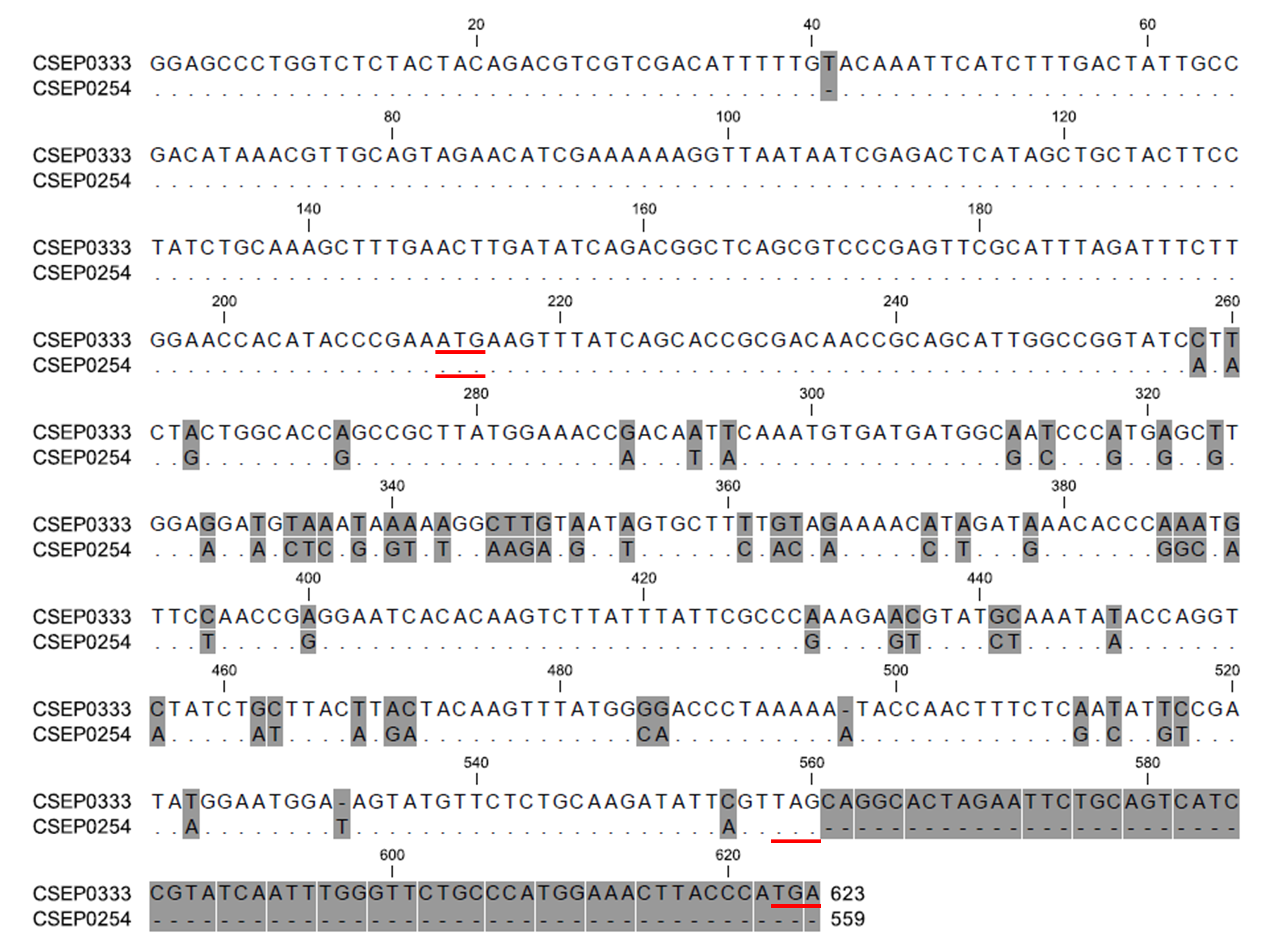

Supplement: S2 Fig — Nucleotides 1–212 belong to the 5’ untranslated region. The remaining are coding sequences. Dots and shades indicate identical and different nucleotides, respectively. Underlined nucleotides are start and stop codons. (TIF) [file pone.0157586.s002.tif]

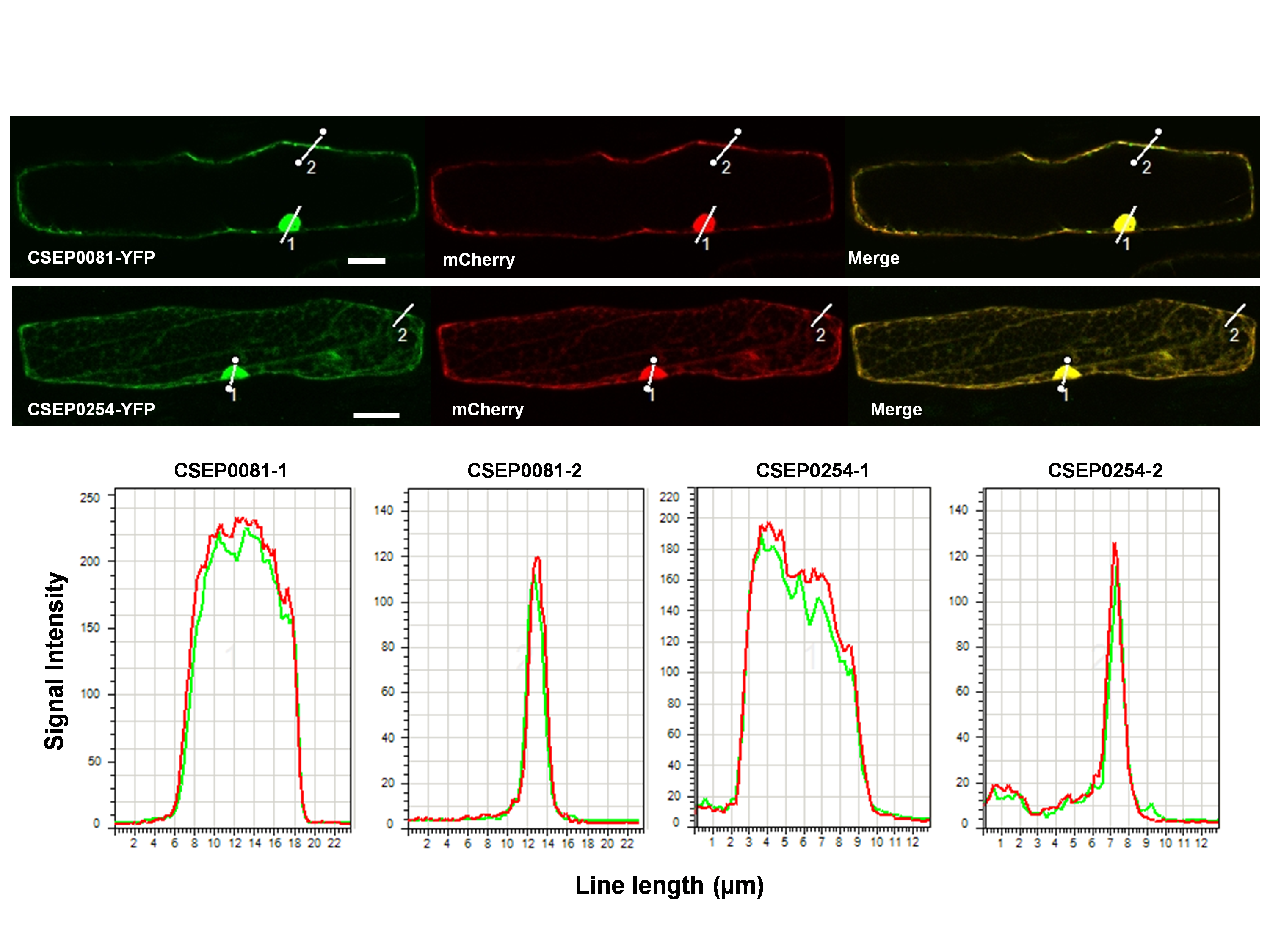

Supplement: S3 Fig — Cross-sectional line was drawn over the nucleus (1) and cytosol (2), and signal was quantified using the Leica microsystems LAS AF image analyzing program. The CSEP-YFP and mCherry signals show overlapping intensity in the nucleus and cytosol. Scale bar, 20 μm. (TIF) [file pone.0157586.s003.tif]
