# Supplementary material for: The Barley Powdery Mildew Effector Candidates CSEP0081 and CSEP0254 Promote Fungal Infection Success
Source: PLoS One. 2016 Jun 20;11(6):e0157586. doi: 10.1371/journal.pone.0157586 (PMC4913928; doi:10.1371/journal.pone.0157586)
Supplement: S1 Table — (DOCX) [file pone.0157586.s004.docx]

**S1 Table. Haustoria count and statistical analysis of HIGS.**

|  | **Number** | **Number of cells** | | **Relative** |  | **95% conf. limit** | |  |
| --- | --- | --- | --- | --- | --- | --- | --- | --- |
| **RNAi** | **of** | **Containing** |  | **haustorial** | **Stand.** |  |  | **P-value** |
| **constructs** | **experiments** | **haustoria** | **Total** | **formation** | **error** | **Lower** | **Upper** |  |
|  | **(leaves)** |  |  | **rate (%)** |  |  |  |  |
| Empty vector | 5 (34) | 370 | 1821 | 100 | 12 | 0.0000 | 0.0000 | . |
| CSEP0145 | 3 (20) | 48 | 549 | 98 | 23 | -1.1742 | 0.1786 | 0.1492 |
| CSEP0062 | 5 (34) | 370 | 2192 | 86 | 14 | -0.5103 | 0.1516 | 0.2881 |
| CSEP0216 | 3 (20) | 67 | 572 | 82 | 20 | -0.9648 | 0.2417 | 0.2401 |
| CSEP0398 | 3 (20) | 77 | 644 | 76 | 16 | -0.7900 | 0.3619 | 0.4664 |
| CSEP0222 | 3 (20) | 69 | 680 | 72 | 14 | -1.0659 | 0.1130 | 0.1131 |
| CSEP0081 | 5 (34) | 266 | 2068 | 68 | 10 | -1.0110 | -0.2964 | 0.0003 |
| CSEP0254 | 5 (34) | 187 | 1931 | 65 | 10 | -1.2687 | -0.4903 | <0.0001 |
| Mlo | 3 (20) | 39 | 617 | 42 | 8 | -1.6837 | -0.2371 | 0.0093 |
