# Supplementary material for: The Barley Powdery Mildew Effector Candidates CSEP0081 and CSEP0254 Promote Fungal Infection Success
Source: PLoS One. 2016 Jun 20;11(6):e0157586. doi: 10.1371/journal.pone.0157586 (PMC4913928; doi:10.1371/journal.pone.0157586)
Supplement: S2 Table — (DOCX) [file pone.0157586.s005.docx]

**S2 Table. Normalized RT-qPCR CSEP transcript quantification.**

| **Time-** | **CSEP0081** | |  | **CSEP0254** | |
| --- | --- | --- | --- | --- | --- |
| **point^1^** | **Mean^2^** | **S.E.^3^** |  | **Mean** | **S.E.** |
| 0 | 0.0440 | 0.0303 |  | 0.0340 | 0.0185 |
| 3 | 0.0122 | 0.0031 |  | 0.0152 | 0.0040 |
| 6 | 0.0260 | 0.0105 |  | 0.0277 | 0.0101 |
| 12 | 0.0493 | 0.0042 |  | 0.0341 | 0.0021 |
| 24 (H) | 3.1500** | 0.4279 |  | 0.5243** | 0.0957 |
| 24 (E) | 0.0076 | 0.0006 |  | 0.0037 | 0.0009 |
| 48 (H) | 11.8000*** | 0.4933 |  | 0.2807*** | 0.0208 |
| 48 (E) | 0.0085 | 0.0010 |  | 0.0036 | 0.0011 |

^1^Hours post inoculation. First four time-points, fungal tissue sample together with leaf tissue. H, haustoria sampled in leaf tissue. E, epiphytic fungal tissue sampled separately.

^2^Mean of three biological repeats (each average of two technical repeats) normalized relative to the GAPDH transcript.

^3^Standard error of biological repeats.

Statistical analysis of biological repeats: *t* test relative to the 0 h time-point; **, P<0.01; ***, P<0.001.
