# Supplementary material for: The Barley Powdery Mildew Effector Candidates CSEP0081 and CSEP0254 Promote Fungal Infection Success
Source: PLoS One. 2016 Jun 20;11(6):e0157586. doi: 10.1371/journal.pone.0157586 (PMC4913928; doi:10.1371/journal.pone.0157586)
Supplement: S3 Table — (DOCX) [file pone.0157586.s006.docx]

**S3 Table. List of forward (F) and reverse (R) primers for plasmid construction and gene expression assay (5´ to 3´).**

| **Primers for the RNAi constructs** | |
| --- | --- |
| CSEP0062_RNAi_F | CTAGCCAAAAGCAACTACGTGTG |
| CSEP0062_RNAi_R | CATAGGAGAGATCGTCTTCTTCGT |
| CSEP0081_RNAi_F | CGTGAATAGTGGAAATGCAAAGTA |
| CSEP0081_RNAi_R | GGCAGTACATGGTAAATAGCCTTC |
| CSEP0145_RNAi_F | AGCACTCCTTTAACTGTAGCACTG |
| CSEP0145_RNAi_R | CTGCCTCTATTTGTAGATCCCTGT |
| CSEP0216_RNAi_F | ACTTCAAAAGTCCATCAATGTCAA |
| CSEP0216_RNAi_R | ACATCACTTATGCGTCCTGAAGTA |
| CSEP0222_RNAi_F | CAGATTATGAGGTGGAATTTGACA |
| CSEP0222_RNAi_R | TCAGTAATTAGTCGGTTCCTTTCC |
| CSEP0254_RNAi_F | ATTGGCCGGTATCATACTGCT |
| CSEP0254_RNAi_R | TGCAGAGAACATACTATCCATTCC |
| CSEP0398_RNAi_F | CGTACCCTTAATCTTCTCGGTCTA |
| CSEP0398_RNAi_R | CTTTGCAGATCGTATACTGGTCAC |
| **Primers for the localization constructs** | |
| CSEP0081_F | CACCGGAAATGCAAAGTACAATTG |
| CSEP0081_R_without stop codon | CATGGCAGTACATGGTAAATAGCCT |
| CSEP0254_F | CACCAACCAACATTACAAATGTGATGA |
| CSEP0254_R_without stop codon | AAAACTAATATCTTGCAGAGAACATACTAT |
| **Primers for qPCR** | |
| CSEP0081_qPCR_F | TACGCAGGTGGAGTTGTGT |
| CSEP0081_qPCR_R | GCAGTACATGGTAAATAGCCTTCA |
| CSEP0254_qPCR_F | CTTGATGAACACCCGGCTA |
| CSEP0254_qPCR_R | ACCTGGTTTATTTAGATACACTCTTCG |
| *Bgh*GAPDH_qPCR_F | ATGAACTACAAGGCATCCTGTCA |
| *Bgh*GAPDH_qPCR_R | TACCATGCGACTAGCTTAACAAAG |
